# Epidemiological associations between iron and cardiovascular disease and diabetes

**DOI:** 10.3389/fphar.2014.00117

**Published:** 2014-05-20

**Authors:** Debargha Basuli, Richard G. Stevens, Frank M. Torti, Suzy V. Torti

**Affiliations:** ^1^Molecular Biology and Biophysicis, University of Connecticut Health Center, FarmingtonCT, USA; ^2^Division of Epidemiology and Biostatistics, Department of Community Medicine and Health Care, University of Connecticut Health Center, FarmingtonCT, USA; ^3^Internal Medicine, University of Connecticut Health Center, FarmingtonCT, USA

**Keywords:** iron, cardiovascular disease, diabetes mellitus, metabolic syndrome, epidemiologic studies

## Abstract

Disruptions in iron homeostasis are linked to a broad spectrum of chronic conditions including cardiovascular, malignant, metabolic, and neurodegenerative disease. Evidence supporting this contention derives from a variety of analytical approaches, ranging from molecular to population-based studies. This review focuses on key epidemiological studies that assess the relationship between body iron status and chronic diseases, with particular emphasis on atherosclerosis ,metabolic syndrome and diabetes. Multiple surrogates have been used to measure body iron status, including serum ferritin, transferrin saturation, serum iron, and dietary iron intake. The lack of a uniform and standardized means of assessing body iron status has limited the precision of epidemiological associations. Intervention studies using depletion of iron to alter risk have been conducted. Genetic and molecular techniques have helped to explicate the biochemistry of iron metabolism at the molecular level. Plausible explanations for how iron contributes to the pathogenesis of these chronic diseases are beginning to be elucidated. Most evidence supports the hypothesis that excess iron contributes to chronic disease by fostering excess production of free radicals. Overall, epidemiological studies, reinforced by basic science experiments, provide a strong line of evidence supporting the association between iron and elevated risk of cardiovascular disease and diabetes. In this narrative review we attempt to condense the information from existing literature on this topic.

## INTRODUCTION

Cardiovascular disease (CVD) and diabetes are major health problems worldwide. In the United States, approximately one in four deaths are due to heart disease, making it the leading cause of death for both men and women (Heron et al., 2009; Heidenreich et al., 2011)^1^. Coronary heart disease is the most common type of heart disease, and costs the US over 100 billion each year (Go et al., 2013). Risk factors include high blood pressure, high LDL cholesterol, smoking, diabetes, and obesity (MMWR Morb Mortal Wkly Rep 60, 2001). Diabetes is itself a significant health problem that is reaching epidemic proportions, with a global prevalence of 382 million people in 2013. It is estimated that by 2035 this will rise to 592 million^2^.

The search for risk factors and methods of prevention for both CVD and diabetes are major efforts of the medical and research community. The role of iron as a risk factor for CVD and diabetes has drawn attention in part due to the concept that it may be a risk factor susceptible to simple dietary modification. Although this is an oversimplification, many (not all) reports suggest that there is indeed an association between iron and both CVD and diabetes, as detailed in this review.

Epidemiological studies have been a powerful tool to probe the association between iron and CVD and diabetes. Several types of study design have been employed. Each of these has its benefits and limitations. Briefly, epidemiological studies can be divided into observational and experimental studies. The difference between an observation and experimental study is that in the latter, an outcome is studied in a population in the absence or presence of an intervention by the investigator. In an observational study, there is no intervention and the investigator simply “observes” and analyses the relationship between exposure and disease outcome. Observational studies include cohort studies, case-control studies and cross sectional studies. A cohort study is an analysis of risk factors where a disease-free study population is identified and followed prospectively over time and a subsequent evaluation is done to find the association between the exposure and disease outcome. While this kind of study can provide strong scientific evidence of an association between risk factors and disease and a temporal framework to assess causality, it is limited by the requirement for a large sample size and long follow-up duration. Often several biases can adulterate the evidence. A case control study on the other hand starts with groups with and without an outcome and evaluates how much a suspected exposure might have contributed to the present outcome status. Thus in comparison to cohort studies, case control studies are relatively quicker to conduct, inexpensive and require comparatively fewer subjects. Cross sectional studies collect and analyze the data on exposure and disease at one specific time point. Such studies cannot evaluate cause and effect relationships since there is no temporal assessment. **Table 1** shows the level of evidence of different types of epidemiological studies. In the hierarchy of evidence-based medicine, experimental studies (or more specifically randomized controlled trials) are recognized as level I of scientific evidence. However, the consensus over this has recently changed as observational studies have been reported to be as effective as randomized controlled trials in estimating the impact of medical interventions on disease outcomes (Benson and Hartz, 2000; Concato et al., 2000). Of course, it must also be emphasized that for a potential hazard, such as elevated body iron, a randomized controlled trials cannot be performed ethically, although it could be done for a study of iron reduction by, for example, phlebotomy.

**Table 1:** Evidence level provided by epidemiological studies of different design.

Level of evidence	Qualifying studies
I	High quality, multicenter or single center, randomized controlled trial with adequate power: or systemic review of these studies
II	Lesser quality, randomized controlled trial; prospective cohort study; or systemic review of these studies
III	Retrospective comparative study; case-control study; or systemic review of these studies
IV	Case series
V	Expert opinion; case report or clinical example; or evidence based on physiology, bench research, or “first principles”

*Song and Chung (2010).*

Virtually all of these epidemiological analyses have been used to probe the relationship between iron and CVD or diabetes. PubMed searches using the terms “iron heart disease epidemiology” or “iron diabetes epidemiology” identify over 500 papers for each search term. In this narrative review, we have not attempted to be comprehensive, but to focus on key epidemiological studies that have investigated these issues. We provide some historical context, but emphasize recent, well-controlled studies with large sample size.

## IRON AND CARDIOVASCULAR DISEASE

Cardiovascular disease is a broad term that includes ischemic and non-ischemic irregularities. Association with iron has been mainly studied and found in ischemic cardiovascular diseases caused by atherosclerosis. To measure ischemic disease outcome, several different endpoints have been used, including coronary heart disease (CHD), carotid artery plaque formation, coronary artery calcium deposition, carotid intima thickness, and atherosclerosis. CHD has been measured by myocardial infarction and cardiogenic angina occurrences and deaths from such incidents. The role of iron in CVD has generally been explored in a group of individuals using one of these defined endpoints. For the purposes of this review, we have included all of these clinical entities under the umbrella of CVD and have not attempted to differentiate among them.

The most common measurement used in the assessment of body iron has been serum ferritin. Serum ferritin was shown to correlate with body iron stores in the 1970s, and is still used clinically for this purpose (Jacobs et al., 1972; Cook et al., 1974; Jacobs and Worwood, 1975; Wang et al., 2010). However, serum ferritin can also be elevated by acute and chronic inflammation (Wang et al., 2010). Accounting for the contribution of these variables thus becomes an important component of studies that use serum ferritin as a measure of body iron, as discussed below. Less frequently, the ratio of soluble transferrin receptor to ferritin has been used, as it has been suggested that this is a more precise measure for body iron store than ferritin alone (Skikne et al., 1990). Catalytically available iron has also been measured in some studies, with the goal of measuring reactive rather than total iron. This approach derives from the consideration that the preponderance of iron in the body is bound to proteins and is not available for participation in the potentially deleterious reactions that are thought to underlie much of the toxicity of iron, such as the formation of reactive oxygen species. A limitation of this approach is that since catalytically available iron represents a relatively small fraction of total iron, its measurement is technically challenging.

The hypothesis that iron status could influence the risk of coronary heart disease was first proposed by Sullivan in the 1980s. Sullivan hypothesized that the higher occurrence of CHD in men and post-menopausal women than in pre-menopausal women was due to higher iron stores in them compared to menstruating women (Sullivan, 1981, 1989). Some earlier studies supported the hypothesis. In a cohort of 2873 Framingham women, an increase in incidence of CHD and disease severity was observed in women who had either natural or surgical menopause (Gordon et al., 1978). In some early prospective studies, a weak association between high blood hemoglobin and hematocrit and risk of CHD was noted (Cullen et al., 1981; Bottiger and Carlson, 1982; Knottnerus et al., 1988). Hemoglobin and hematocrit are not good surrogates for body iron status and during this period serum ferritin was emerging as the best measurement of body iron status (Cook et al., 1974; Kaltwasser and Werner, 1989). The first report in humans on the association between serum ferritin and CHD risk was published in Salonen et al. (1992a). In this cohort of randomly selected 1931 Eastern Finnish men, serum ferritin concentration had a significant association with ischemic heart disease risk. Subjects with serum ferritin ≥200 μg/l had a 2.2-fold (95% CI, 1.2–4.0; *p* < 0.01) higher risk of acute myocardial infarction compared to men with lower serum ferritin. Total blood leucocyte count was adjusted in the statistical analysis to rule out the potential confounding effect of inflammation or chronic vascular disease that would elevate serum ferritin independent of body iron status. The association was stronger in men with higher concentrations of low density lipoproteins (RR = 1.8, 95% CI, 0.9–3.5, NS in men with low LDL and a RR = 4.7, 95% CI, 1.4–16.3, *p* < 0.05 in men with high LDL). After this report, the group conducted another nested case-control study within the Kuopio Ischemic Heart Disease Risk Factor Study (KIHD) cohort and found that men with high body iron stores were at increased risk of acute myocardial infarction (AMI), confirming their original observation (Tuomainen et al., 1998). In this study body iron status was measured by ratio of soluble TfR and ferritin which some authors suggest as a better measure of body iron than serum ferritin alone (Cook et al., 1974; Skikne et al., 1990).

The first prospective study in women was conducted in 11,471 Dutch post-menopausal female subjects aged 49–70 years (van der et al., 2005). In the study, the multivariate hazard ratio of ischemic strokes in the highest tertile of serum ferritin concentration was 2.23(95% CI, 1.05–4.73) compared to the lowest. An interesting finding common to some of these studies was the interaction of LDL with serum ferritin in increasing the risk of ischemic events. A plausible biological mechanism underlying this interaction may be the ability of iron to produce reactive oxygen species. Iron catalyzes the Fenton reaction which produces potent oxidants that increase the risk of atherosclerosis by promoting the peroxidation of lipids (McCord, 1991; Salonen et al., 1992b; Berliner and Heinecke, 1996). Local release of iron from ferritin by superoxide radical generated by ischemia/reperfusion injury to blood vessels may further exacerbate this damage (Thomas et al., 1985).

Many of the studies discussed above focused on cardiovascular events such as acute myocardial infarction. However, myocardial infarction is a complex endpoint resulting from multiple potential pathogenesis pathways. To circumvent this limitation, other studies used preclinical atherosclerosis as the dependent variable and explored its relationship to serum ferritin. For example, Kiechl et al. (1997) reported that serum ferritin level was closely related to incidence of carotid atherosclerosis and progression of previous atherosclerotic lesions in a cohort of Italian men and women. In a cross sectional study that included German men and women, there was an association of serum ferritin with carotid plaque prevalence in both men (OR: 1.33; 95% CI, 1.08–1.44) and women (OR, 1.29; 95% CI, 0.98–1.75) (Wolff et al., 2004). When the study population was divided into ferritin octiles, both men and women showed a dose-dependent relationship between serum ferritin and atherosclerotic plaques. Subjects with malignancy and liver diseases were excluded to eliminate the confounding effect of inflammation and mild liver disease, but no adjustments were made for any inflammatory markers. Thus, acute or chronic inflammatory conditions could have confounded the findings in this study by affecting serum ferritin levels at the time of measurement.

Several recent studies have shown that serum ferritin is independently associated with preclinical measures of vascular diseases. Sung et al. (2012) showed that ferritin levels in a large cohort of 12,033 young Korean men were independently associated with coronary artery calcium content, a marker of early coronary artery sclerosis. In a similar study, Valenti et al. (2011) showed that in a small study population of non-alcoholic fatty liver patients, carotid intima medial thickness and carotid plaque were independently associated with increased ferritin levels.

The potentially damaging effect of iron on the heart, pancreas, liver and other organs was made evident in part through the study of hemochromatosis, a disorder in which excess iron is absorbed and deposited in tissues. Patients with untreated hemochromatosis can exhibit diabetes, liver damage, and cardiac injury among other symptoms (Wolff, 1993; Witte et al., 1996; Powell et al., 1998). Mutations in HFE gene (the hemochromatosis gene) are one cause of hemochromatosis. Using a mouse model for hereditary hemochromatosis, Turoczi et al. (2003) showed an interaction of dietary iron intake and HFE gene status (KO vs. wildtype) in degree of ischemia/reperfusion injury to heart; HFE KO mice showed greater ventricular dysfunction, myocardial infarct size, and cardiomyocyte apoptosis compared to wild type mice on a standard diet, and an even greater degree of damage in the HFE KO mice fed a high iron diet (Turoczi et al., 2003). In human subjects, a similar increase in cardiovascular death was observed in women heterozygous for the HFE gene (Roest et al., 1999). However, no association between the HFE genotype and atherosclerosis has been found in hemochromatosis patients in spite of iron overload status in these patients (van der et al., 2006; Engberink et al., 2010). Valenti et al. (2011) found that the prevalence of carotid plaques was highest in patients with hyperferritinemia independent of HFE genotype. Although the risk of atherosclerotic heart disease appears unrelated to HFE genotype, hemochromatosis patients do have a higher risk of iron-related non-ischemic cardiovascular irregularities (Gaenzer et al., 2002; Dunn et al., 2008).

Multiple mechanisms likely underlie the association of iron with CVD. In addition to the ability of iron to promote lipid peroxidation, recent studies have implicated the peptide hormone hepcidin in atherosclerosis. Hepcidin is a central regulator of iron absorption and recycling (Ganz, 2013). Hepcidin acts by binding ferroportin, an iron efflux pump present in both enterocytes and macrophages. Binding of hepcidin to ferroportin triggers ferroportin degradation (Nemeth et al., 2004), thus inhibiting the delivery of iron to the circulation through the enterocyte as well as inhibiting iron recycling in macrophages. Valenti et al. (2011) observed that serum hepcidin was independently associated with carotid plaques, suggesting that hepcidin-induced iron accumulation may be involved in the process of atherogenesis in subjects negative for HFE mutations. Specifically, hepcidin may induce excessive iron trapping within macrophages, resulting in an increase in oxidative stress, transformation into foam cells, and ultimately to atherosclerotic vascular disease (Sullivan, 2007, 2009; Theurl et al., 2008). This hypothesis is known as the “iron hypothesis” and was proposed by Sullivan (2009). Since hepcidin is decreased in patients with hereditary hemochromatosis, this model provides a potential explanation for the previous observation that atherosclerosis risk is not increased in subjects with hereditary hemochromatosis (van der et al., 2006). However, a recent experimental study provided evidence that hepatic hepcidin expression is not correlated with atherosclerosis progression in a mouse model Kautz et al. (2013). Further, the authors reported that increasing macrophage iron accumulation in mice with atherosclerosis either through a genetic mutation in the ferroportin gene or through parenteral iron administration failed to increase the size of atherosclerotic lesions or lesion calcification. The study challenged the “iron hypothesis.”

An alternative approach to the use of serum ferritin to assess the relationship between iron and CVD has been to assess the relationship between catalytic iron and heart disease. Catalytic iron is the iron that is not bound to transferrin or ferritin and is available to take part in chemical reactions to produce oxidant products. This can be measured using a bleomycin detectable iron assay (BDI; von Bonsdorff et al., 2002). Results from such studies are equivocal. While some studies found a relation between catalytic iron with CVDs (Rajapurkar et al., 2012), a recent study with a larger study population failed to report any association of catalytic iron with risk of MI and recurrent ischemic events (Steen et al., 2013). However the study showed that in a cohort of 1701 patients with AMI or unstable angina, catalytic iron was associated with stepwise increase in all-cause mortality (multivariate adjusted HR = 3.97, 95% CI 1.09–14.1, *p* = 0.036, highest quartile vs. baseline) when followed for a median of 10 months. Although no significant association of ischemic events with iron was reported, most of the deaths were related to ischemic complications, and thus the contribution of catalytic iron could not be ruled out completely. It is to be noted that serum catalytic iron does not reflect the intraplaque iron which might be a more proximal factor for vascular ischemic injuries (Nelson et al., 1992; Castellanos et al., 2002). Unfortunately, these studies did not report the relationship between serum ferritin and outcome. It thus remains unaddressed whether or not catalytic iron is more strongly associated than serum ferritin with outcome.

Another approach to testing a potential link between iron and CVDs has been the study of dietary iron intake and risk. Zhang et al. (2012) reported that dietary intake of total iron was positively associated with deaths from strokes and CVD in a cohort of 23,083 Asian men with a multivariate hazard ratio of 1.43 (95% CI, 1.02–2, *p* = 0.009) for stroke and 1.27 (95% CI, 1.01–1.58, *p* = 0.023) for CVD after adjustment for other CVD risk factors; iron intake was not associated with these outcomes in women, however. In another large prospective study in almost 50,000 European men, there was a positive association of dietary iron, more specifically heme iron (a form of iron that is more readily absorbed by the gut than inorganic iron), with strokes in a follow-up period of 11.7 years (Kaluza et al., 2013). Adjustments were made for red meat consumption to rule out confounding by other known risk factors for stroke such as *N*-nitroso compounds and heterocyclic compounds (Forstermann, 2008). The association was observed in normal weight men and not in overweight or obese men, most likely because of decreased iron absorption due to increased hepcidin in the chronic inflammatory state associated with obesity (Greenberg and Obin, 2006). Adipocyte hepcidin expression is known to be positively correlated with obesity (Bekri et al., 2006)^.^ This association is still to be properly evaluated in women.

Although the foregoing studies appear well-conducted, there are also a number of other well-conducted studies that have found no association of markers of body iron and risk of CVD (Baer et al., 1994; Danesh and Appleby, 1999; Gupta et al., 2000; Knuiman et al., 2003; Sun et al., 2008b; Friedrich et al., 2009). Discordant results among studies may in part be due to imprecision in the surrogate markers used to measure iron status (ferritin, total iron binding capacity, transferrin saturation, serum iron, and dietary iron intake), which are all indirect measures of body iron stores. Because these variables are subject to temporal and measurement variations, there is undoubtedly exposure misclassification in the subjects. This non-differential misclassification would reduce the ability of a study to identify a true association should one exist. An additional potential problem, particularly in cross-sectional studies, is that observed elevations in serum ferritin may represent an effect rather than a cause of underlying pathology.

If iron is associated with CVD, can interventions that reduce iron reduce risk? Unfortunately, only a limited number of intervention studies have been conducted, but results of these studies are at least suggestive that modulating iron can reduce risk. In animal models, treatment with deferoxamine, an iron chelator, during ischemia improved recovery and reduced reperfusion-induced oxygen radical formation in rabbit hearts (Williams et al., 1991). Paraskevaidis et al. (2005) reported that deferoxamine infusion ameliorated lipid peroxidation and improved long term outcome in patients having coronary artery bypass surgery. In a recent randomized controlled single blinded study, Zacharski et al. (2011) showed that a lower ferritin level predicted improved outcome and iron reduction by phlebotomy improved outcomes by preventing or delaying non-fatal myocardial infarction and stroke in young age patients with peripheral artery disease.

## IRON AND METABOLIC SYNDROME AND DIABETES

Metabolic syndrome refers to a collection of risk factors that increase the likelihood of heart disease, diabetes and stroke. They include a large waistline, high triglyceride, low HDL cholesterol, high blood pressure and high fasting blood sugar. The presence of three of these five risk factors, many of which are associated with obesity, is defined as metabolic syndrome^3^.

Multiple studies have shown that excess body iron is associated with one or more components of metabolic syndrome (Jehn et al., 2004; Bozzini et al., 2005; Choi et al., 2005; Gonzalez et al., 2006; Sun et al., 2008a). To study the association of iron with metabolic syndrome in normal individuals, a cross-sectional study in 6044 US adults was conducted. The results showed a significant association of ferritin level with metabolic syndrome and insulin resistance (IR) after excluding hemochromatosis cases and adjusting for age, race/ethnicity, C-reactive protein, smoking, alcohol intake, and BMI (Jehn et al., 2004). Other studies in western populations showed similar associations (Jehn et al., 2004; Bozzini et al., 2005; Gonzalez et al., 2006; Wrede et al., 2006). A positive association of serum ferritin with the prevalence of metabolic syndrome in a study population of 8441 people including both sexes and from different provinces of China was recently reported (Li et al., 2013). Mendler et al. (1999) showed that in a cohort of patients with unexplained hepatic iron overload, IR was also often observed. Such patients with non-alcoholic fatty liver disease also tend to have elevated ferritin levels (Valenti et al., 2003, 2006, 2007, 2010) which is now considered a feature of metabolic syndrome (Marchesini et al., 2001, 2003; Angulo, 2002). The constellation of hepatic steatosis, mild to moderate iron overload in both hepatocytes and macrophages, increased serum ferritin levels, and insulin resistance is commonly referred to as dysmetabolic iron overload syndrome or DIOS (Barisani et al., 2008; Riva et al., 2008; Datz et al., 2013). DIOS is detected in about one third of the patients with NAFLD and MetS and may be predisposing factor to the development of type 2 DM and CVDs [Dongiovanni et al., 2011; Valenti et al., 2012; See Dongiovanni et al. (2011) and Datz et al. (2013) for detailed discussion and review of this topic]. Iron depletion in such patients has been shown to improve histological liver damage and abnormal liver function when compared to lifestyle modification (Valenti et al., 2014).

Similarly, many epidemiological studies have reported statistically significant associations of body iron with diabetes, although results from all studies are not entirely consistent. In one longitudinal study of overweight/obese individuals with an impaired glucose tolerance test, there was no association between ferritin levels with risk of diabetes (Rajpathak et al., 2009). In another similar study, adjustment for BMI and components of the metabolic syndrome produced a null result (Jehn et al., 2007). However, in a prospective study in China, a country with the largest diabetic population in the world, an almost twofold increased risk of type 2 diabetes was observed among middle aged and elderly persons in the highest quintile of ferritin level compared with those in lowest after adjusting for known risk factors including high sensitivity C reactive protein (hsCRP), BMI, γ-glutamyl transferase (GGT), and adiponectin (Sun et al., 2013). The result was consistent with findings from similar prospective cohort studies in Caucasian populations (Salonen et al., 1998; Jiang et al., 2004; Forouhi et al., 2007; Montonen et al., 2012). In the EPIC (European Prospective Investigation of Cancer)-Norfolk cohort, serum ferritin was an important and independent predicting factor for development of diabetes after adjustment for conventional risk factors as well as vitamin C levels, CRP, IL6, liver function test (ALT, GGT), fibrinogen and adiponectin (Forouhi et al., 2007). In a case cohort study among 27,548 participants of the EPIC Postdam study in Germany, the sTFR-ferritin ratio was significantly inversely related to the risk of type 2 diabetes, and ferritin concentration was associated with higher risk (Montonen et al., 2012). The result was independent of biomarkers of inflammation, hepatic fat, IR, and dyslipidemia.

In evaluating these studies, a few considerations must be borne in mind. In epidemiological studies investigating the relation between iron and diabetes, serum ferritin is the most commonly used indicator of body iron stores. As mentioned in the introduction, the use of ferritin in assessing body iron stores has been somewhat challenging because ferritin can be elevated in inflammation, cancer, and liver disease (Wang et al., 2010). Serum ferritin concentration can also be increased in some conditions like obesity and metabolic syndrome which are associated risk factors for type 2 diabetes (Lee et al., 2009). It thus becomes difficult to discern whether the association of ferritin with diabetes is due to other concomitant conditions or serum ferritin levels increase as a result of diabetes (a case of reverse causation). In addition, serum ferritin has been correlated with dyslipidemia biomarkers (Halle et al., 1997), hepatic enzymes (Choi et al., 2005), and negatively associated with adiponectin, an insulin sensitizing adipokine that is decreased in diabetic patients (Forouhi et al., 2007). Thus adjustments for these components become very important.

The exact molecular mechanism of iron-related pathology in metabolic syndrome and diabetes is not clearly understood. Iron is a powerful pro-oxidant and can cause cellular damage by producing reactive oxygen species in different tissues of the body (Andrews, 1999; Rajpathak et al., 2009). Insulin producing pancreatic β cells have been shown to be particularly susceptible to oxidative injury, in part due to decreased expression of antioxidant enzymes such as dismutase, catalase, and glutathione peroxidase (Tiedge et al., 1997). Thus iron deposition in β cells can lead to apoptosis and consequently to decreased insulin synthesis and secretion (Tiedge et al., 1997; Ferrannini, 2000; Cooksey et al., 2004). A recent study in a mouse model of iron overload showed that iron deposition enhances fatty acid oxidation and decreases glucose oxidation in skeletal muscle by inhibiting pyruvate dehydrogenase (PDH) enzyme activity thus increasing IR (Huang et al., 2011). Glucose oxidation is decreased in adipose tissue (Merkel et al., 1988; Green et al., 2006). Iron accumulation also results in an abnormal increase in hepatic glucose production (Mendler et al., 1999; Ferrannini, 2000; Green et al., 2006), inappropriate hepatic insulin extraction, and affects insulin secretion in the pancreas (Niederau et al., 1984). A recent study of 492 subjects demonstrated an association between markers of iron metabolism, adipocyte insulin resistance, and adiponectin (an insulin-sensitizing adipokine), consistent with a model in which iron contributes to T2DM by inducing insulin resistance in adipocytes (Wlazlo et al., 2013). Consistent with this model, mice fed a high iron diet exhibited an accumulation of iron within adipocytes and altered transcription of adipokines involved in glycemic control (Gabrielsen et al., 2012; Dongiovanni et al., 2013). In particular, iron downregulated adiponectin (an adipokine with insulin sensitizing action; Gabrielsen et al., 2012) and increased resistin (an adipokine with hyperglycemic action; Dongiovanni et al., 2013).

The association of dietary iron and diabetes has also been examined. Iron in the diet exists as heme (organic) and non-heme (inorganic) forms. Some studies have shown that the risk of diabetes can be increased by heme iron in the diet (Jiang et al., 2004; Lee et al., 2004; Rajpathak et al., 2006)^.^ Most of these studies were conducted in a healthy US population. In Asian populations, a similar association was reported in a cross sectional study of 2997 Chinese people (Luan de et al., 2008). Consistent results were obtained in an observational cohort of Mediterranean people (Fernandez-Cao et al., 2013). Two recent meta-analyses concluded that higher heme iron poses higher risk of type 2 diabetes (Bao et al., 2012; Zhao et al., 2012). One of the studies reported that there was no significant association with total iron, non-heme iron or iron supplements in the diet (Bao et al., 2012). However, these studies did not separate heme iron *per se* from other components of red meat. Red meat bears a high correlation with heme iron and has been shown to be associated with the risk of type 2 diabetes (Pan et al., 2011). Therefore confounding by other components from red meat cannot be ruled out with certainty. Screening for HFE mutation was not conducted in these studies, and hence a contribution of genetic interaction cannot be ruled out either.

Despite the limitations of epidemiological studies, intervention studies support the association between excess iron, metabolic syndrome and diabetes. Iron reduction by phlebotomy and chelation therapy produced an improvement in glucose tolerance not only in patients with hemochromatosis (Dymock et al., 1972; Inoue et al., 1997), but also in healthy donors. Houschyar et al. (2012) reported that reduction of body iron stores through phlebotomy had therapeutic effects in metabolic syndrome patients, including lowered blood pressure and improvement in glycemic control and cardiovascular risks. Fernandez-Real et al. (2002) found that bloodletting in high ferritin type 2 diabetes patients resulted in decrease in glycated hemoglobin and also improved insulin secretion and sensitivity.

## CONCLUSION

**FIGURE 1:**
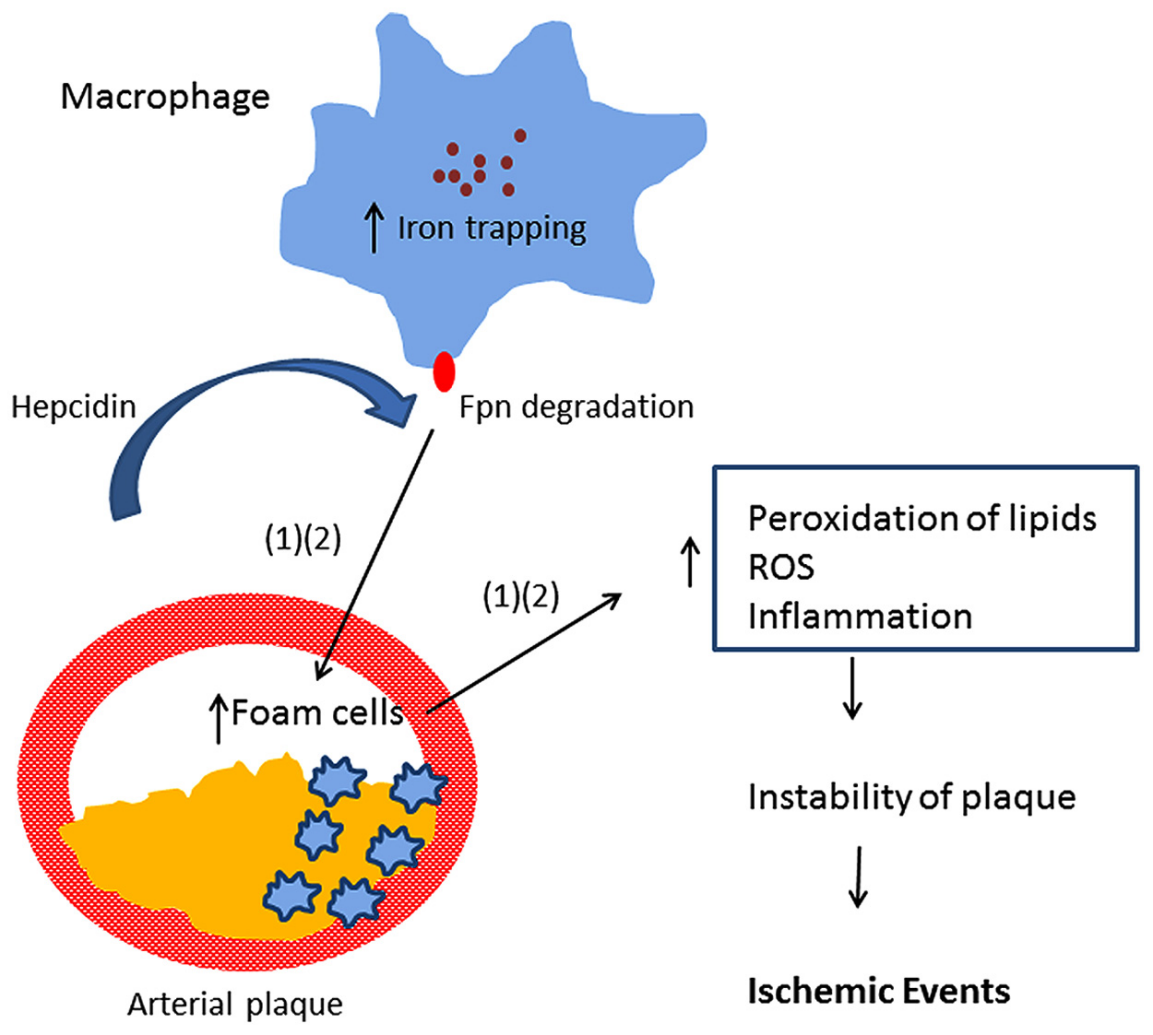
**Model showing iron retention in macrophages promotes arterial plaque destabilization (Sullivan, 2007; Theurl et al., 2008)**.

**FIGURE 2:**
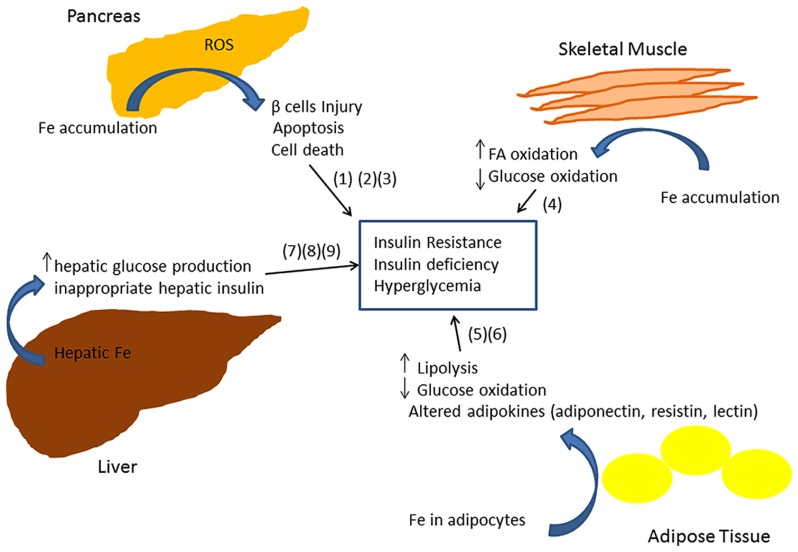
**Model showing multiple mechanisms through which iron can lead to insulin resistance and insufficiency (Merkel et al., 1988; Tiedge et al., 1997; Mendler et al., 1999; Ferrannini, 2000; Cooksey et al., 2004; Green et al., 2006; Huang et al., 2011)**.

Epidemiological studies provide evidence that elevated iron stores are a risk factor for developing cardiovascular and metabolic abnormalities. Such results have been verified in diverse ethnic and geographic populations. Although mechanistic insights have been limited, iron-dependent pathophysiological pathways involved in these two conditions may exhibit some differences. In diabetes and metabolic syndrome, iron may contribute to risk following deposition in the liver, pancreas, and skeletal muscle, where it can enhance oxidative damage and contribute to insulin deficiency and resistance. In CVD, iron within macrophages and foam cells predisposes to the formation of atherosclerotic plaques. Hepcidin may promote plaque destabilization by preventing iron export from the intralesional macrophages leading to ischemic events. Although additional mechanisms are likely involved, **Figures 1** and **2** illustrate some pathways through which excess iron can increase risk of CVD, metabolic syndrome, and diabetes.

Regarding iron in the diet, there is still insufficient data to formulate guidelines on dietary iron restrictions in the at-risk or general population. This is primarily because dietary iron exists in two very different forms – heme and non-heme iron. Study findings are more inclined toward the association of heme iron (mainly from meat) and disease risk rather than non-heme iron. Although some studies have attempted to assess whether iron supplementation is linked to disease risk, particularly diabetes in women, the results have been inconsistent (Rajpathak et al., 2006; Bo et al., 2009; Chan et al., 2009).

Further research is required to identify more predictors of body iron stores that may help in reducing the risk of cardiovascular or metabolic disease. Experiments are needed to unravel the underlying biological mechanism of this association. Additionally, more randomized controlled studies are warranted to evaluate the clinical outcome of patients placed on iron restricted diets or subjected to iron depletion therapy so that therapeutic recommendations can be made.

## Conflict of Interest Statement

The authors declare that the research was conducted in the absence of any commercial or financial relationships that could be construed as a potential conflict of interest.

## Footnotes

1 http://www.cdc.gov/heartdisease/facts.htm (accessed February 2, 2014).

2 http://www.idf.org/diabetesatlas

3 http://www.nhlbi.nih.gov/health/health-topics/topics/ms/
